# The Role of Muscarinic Receptors in the Beneficial Effects of Adenosine against Myocardial Reperfusion Injury in Rats

**DOI:** 10.1371/journal.pone.0025618

**Published:** 2011-11-04

**Authors:** Lei Sun, Dong-Ling Li, Mei Zhao, Xi He, Xiao-Jiang Yu, Yi Miao, Hao Wang, Jun Ren, Wei-Jin Zang

**Affiliations:** 1 Department of Pharmacology, College of Medicine, Xi'an Jiaotong University, Xi'an, China; 2 Center for Cardiovascular Research and Alternative Medicine, University of Wyoming College of Health Sciences, Laramie, Wyoming, United States of America; University of Colorado Denver, United States of America

## Abstract

Adenosine, a catabolite of ATP, displays a wide variety of effects in the heart including regulation of cardiac response to myocardial ischemia and reperfusion injury. Nonetheless, the precise mechanism of adenosine-induced cardioprotection is still elusive. Isolated Sprague-Dawley rat hearts underwent 30 min global ischemia and 120 min reperfusion using a Langendorff apparatus. Both adenosine and acetylcholine treatment recovered the post-reperfusion cardiac function associated with adenosine and muscarinic receptors activation. Simultaneous administration of adenosine and acetylcholine failed to exert any additive protective effect, suggesting a shared mechanism between the two. Our data further revealed a cross-talk between the adenosine and acetylcholine receptor signaling in reperfused rat hearts. Interestingly, the selective M_2_ muscarinic acetylcholine receptor antagonist methoctramine significantly attenuated the cardioprotective effect of adenosine. In addition, treatment with adenosine upregulated the expression and the maximal binding capacity of muscarinic acetylcholine receptor, which were inhibited by the selective A_1_ adenosine receptor antagonist 8-Cyclopentyl-1,3-dipropylxanthine (DPCPX) and the nitric oxide synthase inhibitor N^ω^-nitro-L-arginine methyl ester (L-NAME). These data suggested a possible functional coupling between the adenosine and muscarinic receptors behind the observed cardioprotection. Furthermore, nitric oxide was found involved in triggering the response to each of the two receptor agonist. In summary, there may be a cross-talk between the adenosine and muscarinic receptors in ischemic/reperfused myocardium with nitric oxide synthase might serve as the distal converging point. In addition, adenosine contributes to the invigorating effect of adenosine on muscarinic receptor thereby prompting to regulation of cardiac function. These findings argue for a potentially novel mechanism behind the adenosine-mediated cardioprotection.

## Introduction

Adenosine is an endogenous purine metabolite that may act as acute “retaliatory” systems mediating immediate responses to injurious stimuli and offer potential as targets for therapeutic cardioprotection. Substantial evidence has accumulated that adenosine is capable of rapidly responding to myocardial ischemic stress and reperfusion insult, contributing to coronary hyperemia, improved microcirculation and reduced infarct size [1]–[4]. Several small-scale clinical studies have recently demonstrated that administration of adenosine during reperfusion rescues ventricular function and improves the overall clinical outcomes [5]. Adenosine mediates various cardiovascular responses via its receptor subtypes (A_1_AR, A_2A_AR, A_2B_AR, and A_3_AR), which are expressed in different cell types in the heart and vessels, and are coupled to different G proteins to trigger a range of physiological responses [6]. Adenosine elicits its cardioprotection via activation of A_1_AR to attenuate myocardial responsiveness to toxic effects of adrenergic overstimulation [3]. Such antiadrenergic property of adenosine plays an essential role in the regulation of autonomic nervous activity.

It is well known that autonomic dysregulation in the heart, which is manifested as suppressed vagal (parasympathetic) activity in combination with increased sympathetic activity, leads to severe cardiovascular sequelae including myocardial ischemia, hypertension, heart failure and arrhythmia [7], [8]. In fact, the decreased expression and impaired function of muscarinic acetylcholine receptor (MAChR) may be responsible for the diminished vagal activity. On the other hand, some studies have shown that beta-blocker (eg. carvedilol) [9] and angiotensin-converting enzyme inhibitors [10] restore vagal tone on heart failure partly by increasing M_2_AChR (the main MAChR in mammalian heart). Our previous study also has indicated that the exogenous precursor of adenosine, namely adenine sulfate exerts cardioprotection via the up-regulation of muscarinic receptor and cholinergic nerve density [11].

Recent reports put forward that adenosine may act in concert with parasympathetic nerve system [12]. In terms of cardiac protection, adenosine infusion enhances cholinergic effects in isolated canine atria [13]. Moreover, Silinsky E and colleagues have shown that adenosine may promote acetylcholine secretion in motor neurons [14]. However, there are few systematic studies with regards to whether and how adenosine regulates parasympathetic nerve system to ameliorate heart function. Therefore, the present study will focus on the interaction between adenosine and muscarinic receptors in the ischemic-reperfused myocardium.

## Materials and Methods

### Ethics Statement

Adult male Sprague-Dawley rats were supplied by the Experimental Animal Center of Xi'an Jiaotong University, China, and weighing 180–220 g. This study was carried out in strict accordance with the Guidelines on the Care and Use of Laboratory Animals issued by the Chinese Council on Animal Research and the Guidelines of Animal Care. The protocol was approved by the ethical committee of Xi'an Jiaotong University. All surgery was performed under sodium pentobarbital anesthesia, and all efforts were made to minimize suffering.

### Langendorff-isolated Perfused Heart Preparation

Rats were sacrificed by cervical dislocation after anesthesia with 3% sodium pentobarbital (40 mg/kg, intraperitoneal injection), and their hearts were excised rapidly and rinsed by immersion in ice-cold Krebs-Henseleit buffer (KHB) (mM: NaCl 118.5, KCl 4.7, MgSO_4_ 1.2, CaCl_2_ 1.8, NaHCO_3_ 25.0, and glucose 11.0 at pH 7.35). Hearts were mounted on a non-recirculating Langendorff apparatus (ML785B2, ADInstruments, Inc., MA, Australia) and retrogradely perfused with warm (37°C), oxygenated (95% O_2_, 5% CO_2_) KHB at a constant pressure of 70 mmHg. The organ chamber temperature was maintained at 37°C during the experiment. A water-filled latex balloon was inserted through an incision in the left atrium into the left ventricle via the mitral valve and adjusted to a left ventricular end-diastolic pressure (LVEDP) of 5–7 mmHg during initial equilibrium. The distal end of the catheter was connected to a PowerLab 8/SP TM data acquisition system (Chart 5.0 software, AD Instruments Inc., MA, Australia) via a pressure transducer for continuous recording. Left ventricular systolic function was assessed by recording the left-ventricular developed pressure (LVDP), which was defined as the difference between left-ventricular systolic pressure (LVSP) and LVEDP. Heart rate and coronary flow rate were monitored simultaneously. In each experiment, after 30 min of stabilization perfused with KHB, hearts of all groups underwent 30 min global ischemia (zero perfusion) and 120 min reperfusion.

### Experimental Protocols

The hearts were randomly assigned to one of the following treatments that were applied for the first 60 minutes of reperfusion: (1) KHB (IR); (2) adenosine (Ado, 0.1 mM); (3) acetylcholine (ACh, 0.1 mM); (4) adenosine+acetylcholine; (5) selective antagonist for the A_1_ adenosine receptor (A_1_AR) 8-Cyclopentyl-1,3-dipropylxanthine (DPCPX, 1.0 µM); (6) selective antagonist for the M_2_ muscarinic acetylcholine receptor (M_2_AChR) methoctramine (METH, 1.0 µM); (7) non-specific nitric oxide synthase (NOS) inhibitor: N^ω^-nitro-L-arginine methyl ester: (L-NAME, 0.1 mM); (8) Ado+DPCPX; (9) ACh+DPCPX; (10) Ado+METH; (11) ACh+METH; (12) Ado+L-NAME and (13) ACh+L-NAME (n = 6 in each group). At the 60th min after the onset of reperfusion, coronary effluent was collected continuously for the lactate dehydrogenase (LDH) and nitric oxide (NO) assay. At the end of reperfusion, each heart was quickly removed and immediately frozen in liquid nitrogen and stored at −80°C for subsequent testing.

### Western Blots

Frozen heart samples collected at the end of reperfusion were transferred to an ice cold RIPA lysis buffer (Beyotime Biotech, Nantong, China) containing phenylmethanesulfonyl fluoride and homogenized with a tissue homogenizer. After centrifugation (12,000 rpm, 5 min at 4°C), the tissue lysates were used in Western blot analyses. Protein concentration was determined using the BCA protein assay kit (Beyotime Biotech). Samples were boiled for 5 min and then loaded on 10% sodium dodecyl sulfate-polyacrylamide gel electrophoresis (SDS-PAGE) and electrotransferred to PVDF-membrane, which was subsequently blocked with 5% skim milk for 1 h at 37°C. Membranes were incubated with primary antibodies directed against anti-β-actin and anti-M_2_ (1∶200; Santa Cruz, CA, USA) in 5% TBST at 4°C overnight. After washing membranes with TBST for three times, membranes were incubated with horseradish-peroxidase-conjugated secondary antibody (1∶2000; Bios, Beijing, China) for 1 h at 37°C. After being washed, protein bands were shown using ECL-Plus reagent (Pierce, Rockford, IL, USA) and quantified by scanning densitometry.

### Radioligand Receptor Binding Assay

Myocardial tissues were finely minced in ice-cold Tris-HCl buffer and then were homogenized at 4°C. After centrifugation, the supernatant was filtered through cheesecloth and then re-centrifuged at 20,000×g for 30 min. The resulting pellet was gently resuspended and samples were removed for protein determination using the Coomassie Brilliant Blue method. Membrane suspensions from either the atrium or ventricles were incubated with increasing concentrations of [^3^H] quinuclidinyl benzilate (QNB 0.1–6 nM, a potent central muscarinic antagonist) to measure total binding. Non-specific binding was defined in the presence of 2 µM atropine. Specific binding was estimated as the difference between total and non-specific binding. Membranes were filtered using a tissue harvester through GF/B filters presoaked in sodium phosphate buffer containing for 12 h. Individual filters were washed three times with ice-cold buffer and dried in an incubator at 80°C. Once dried, 5 mL of scintillation liquid (23 mM PPO, 0.8 mM POPOP, 10% v/v triton X-100 dissolved in toluene) was added. The filters were left overnight before the radioactivity was measured by liquid scintillation spectrometry.

### Determination of LDH and NO_x_

Lactate dehydrogenase (LDH) in coronary effluent was determined colorimetrically at 440 nm using a spectrophotometer by the associated detection kits (Nanjing Jiancheng Bioengineering Institute, NJBI, Niangjing, China). Data were expressed as units of activity per liter coronary effluent (U/L). The index nitric oxide production (NO_x_) in cardiac tissue was determined indirectly as the concentration of nitrite from nitrates by the assay kit (NJBI, Niangjing, China). Briefly, heart tissue was homogenized and centrifuged at 10000×g for 10 min. The supernatant was removed and mixed with equal volume of Griess regent. After incubation for 15 min at room temperature, absorbance was measured spectrophotometrically at 550 nm.

### Statistical Analysis

Values were presented as mean±standard error (SE). Coronary flow rate and cardiac contractility data were analysed using repeated measures analysis of variance ANOVA followed by Newman-Keuls post-hoc test to compare differences in any parameter between groups. Other data were analyzed with a one-way ANOVA followed by a LSD post-hoc test. Significance level was set at *P*<0.05.

## Results

### Effects of Adenosine/Acetylcholine on Cardiac Function and LDH

**Figure 1:**
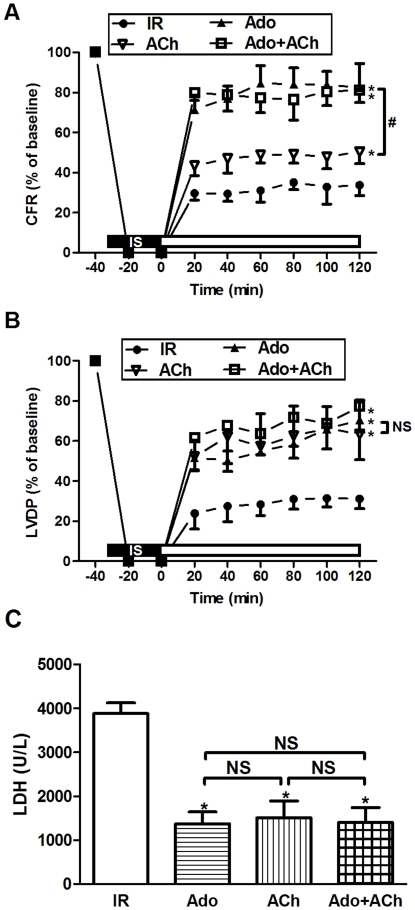
Effects of adenosine/acetylcholine on cardiac function and LDH. (A) Adenosine/acetylcholine improved coronary flow rate (CFR) in ischemic-reperfused rat hearts; (B) Adenosine/acetylcholine improved left-ventricular developed pressure (LVDP) in ischemic-reperfused rat hearts; (C) Adenosine/acetylcholine lessened the LDH release in coronary effluent. IR: ischemic reperfusion; Ado: adenosine; ACh: acetylcholine; IS: iscehmia. ^*^ *P*<0.01 *vs.* IR; ^#^ *P*<0.01 *vs.* Ado; NS: not significance (n = 6 in each group).

Baseline cardiac functional parameters (heart rate, coronary flow rate and LVDP) for various groups are shown in Table 1. No difference was found in the pre-ischemic cardiac function among experimental groups. After 30 min of global ischemia and 120 min of reperfusion, ischemic-reperfused hearts (IR) exhibited a significant reduction in coronary flow rate (CFR) and LVDP (expressed as the percentage of baseline). Both adenosine and acetylcholine treatment recovered the post-reperfusion cardiac function. Adenosine (Ado, 0.1 mM) dramatically improved CFR and LVDP (*P*<0.01 *vs.* IR, Fig. 1A, B). Acetylcholine (ACh, 0.1 mM) produced a slight increase in CFR and marked enhancement in LVDP (*P*<0.01 *vs* IR, Fig. 1A, B). Furthermore, the effect of adenosine on CFR was more pronounced than that of acetylcholine (82.3±4.5% for Ado *vs.* 50.3±6.8% for ACh, *P*<0.01). However, little difference was observed in the improvement of LVDP between Ado and ACh groups (70.5±7.3% for Ado *vs.* 63.3±3.1% for ACh, *P*>0.05). Interestingly, simultaneous administration of both adenosine and acetylcholine failed to exert any additional protective effect over either agonist alone.

**Table 1:** Baseline cardiodynamic data after 30 min of equilibration to Langendorff perfusion.

Group	HR (beat/min)	CFR (mL/min)	LVDP (mmHg)
IR	286±17	12.1±0.8	81.2±14
Ado	292±23	12.6±1.4	89.2±17
ACh	296±23	12.5±0.8	91.0±14
Ado + ACh	289±13	12.4±0.8	89.2±11
Ado + DPCPX	298±19	12.1±1.2	85.8±6
ACh + DPCPX	279±15	12.2±1.1	87.3±6
Ado + METH	300±9	12.2±1.7	90.0±6
ACh + METH	305±15	12.6±1.4	87.8±12
Ado + L-NAME	286±16	12.4±1.5	88.5±17
ACh + L-NAME	300±10	12.8±1.0	84.3±12

Values are means ± SE; n = 6 in each group. HR: heart rate; CFR: coronary flow rate; LVDP: left ventricular developed pressure. There were no significant differences among groups in baseline cardiodynamic data (*P*>0.05).

Myocardial damage was confirmed using LDH in coronary effluent as an indicator of cell cytotoxicity. With the prolongation of reperfusion time, both adenosine and acetylcholine treatment significantly lessened the LDH release (1370±111 for adenosine and 1511±155 for acetylcholine *vs.* 3876±100 for IR, *P*<0.01). When adenosine and acetylcholine were employed in combination, no additive protective effect was observed (Fig. 1C).

### Effects of Adenosine/Acetylcholine and Receptors Blockade on NO_x_ Production

**Figure 2:**
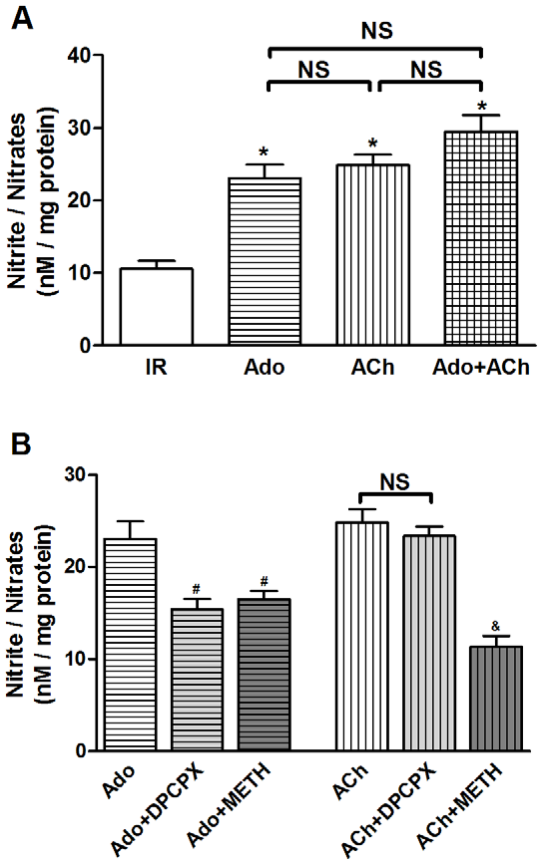
Effects of adenosine/acetylcholine and receptors blockade on NO_x_ production. (A) Adenosine/acetylcholine increased the production of NO_x_ in ischemic-reperfused rat hearts; (B) A_1_AR antagonist DPCPX abolished the increase of NO_x_ afforded by adenosine. M_2_AChR antagonist METH blunted the increase of NO_x_ mediated by adenosine and abolished this effect of acetylcholine. ^*^ *P*<0.01 *vs.* IR; ^#^ *P*<0.01 *vs.* Ado; ^&^ *P*<0.01 *vs.* ACh; NS: not significance (n = 6 in each group).

Treatment with adenosine and acetylcholine resulted in a significant 109% and 123% increase in production of NO_x_ in myocardium compared with the IR group (*P*<0.01 *vs.* IR group), respectively. There was no significant difference between the adenosine and acetylcholine groups. When adenosine and acetylcholine were administered in combination, little additive effect was noted (Fig. 2A). Co-administration of the selective A_1_AR antagonist DPCPX (1.0 µM) and adenosine or the selective M_2_AChR antagonist METH (1.0 µM) and adenosine was performed. DPCPX abolished the increase of NO_x_ afforded by adenosine (*P*<0.01 *vs.* Ado group), but had no such effect on acetylcholine (*P*>0.05 *vs.* ACh group). In the contrary, METH blunted the increase of NO_x_ mediated by adenosine and abolished this effect of acetylcholine (*P*<0.01 *vs.* corresponding control, Fig. 2B). DPCPX and METH alone had no appreciable effect on NO_x_ release (data not shown). These results indicated that the effects of adenosine and acetylcholine on NO_x_ release might likely involve activation of A_1_AR and M_2_AChR, respectively. Interestingly, M_2_AChR seemed to partially mediate the effect of adenosine, and NOS possibly served as the distal common pathway.

### Effects of NOS Inhibitor on Adenosine/Acetylcholine-mediated Cardioprotection

**Figure 3:**
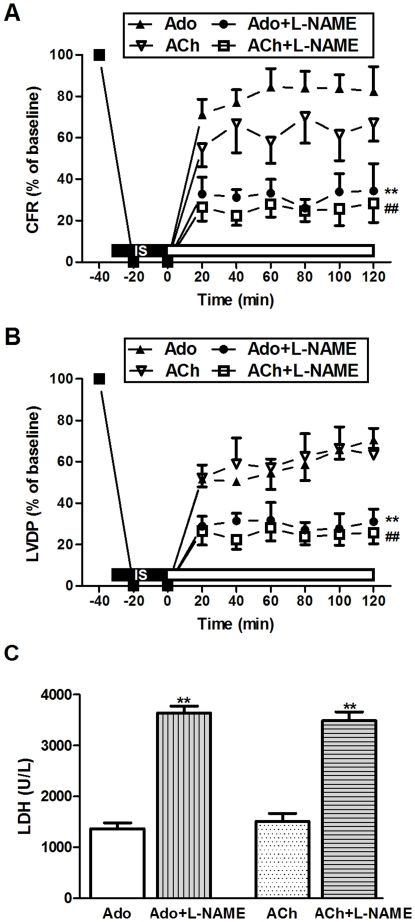
Effects of NOS Inhibitor on adenosine/acetylcholine-mediated cardioprotection. (A) NOS inhibitor L-NAME abolished the increase in coronary flow rate (CFR) mediated by adenosine/acetylcholine; (B) NOS inhibitor L-NAME abolished the left-ventricular developed pressure (LVDP) improvement mediated by adenosine/acetylcholine; (C) NOS inhibitor L-NAME abolished the decrease in LDH release mediated by adenosine/acetylcholine in coronary effluent. Ado: adenosine; ACh: acetylcholine; IS: iscehmia. ^**^ *P*<0.01 *vs.* Ado; ^##^ *P*<0.01 *vs.* ACh (n = 6 in each group).

Co-administration of L-NAME, an inhibitor of NOS and adenosine, L-NAME notably blocked the adenosine-induced improvement in CRF and LVDP (Fig. 3A, B). L-NAME also reversed the inhibitory effect of adenosine on LDH (Fig. 3C). Similarly, Co-administration of L-NAME and acetylcholine, L-NAME abrogated the acetylcholine-induced ameliorative effects in the reperfused hearts. Meanwhile, there were no significant differences in the effects of NOS inhibitor between adenosine and acetylcholine-mediated cardioprotection. Hence, NOS might act in a common pathway to produce cardioprotection afforded by both adenosine and acetylcholine.

### Effects of A_1_AR and M_2_AChR Blockade on Adenosine-mediated Cardioprotection

**Figure 4:**
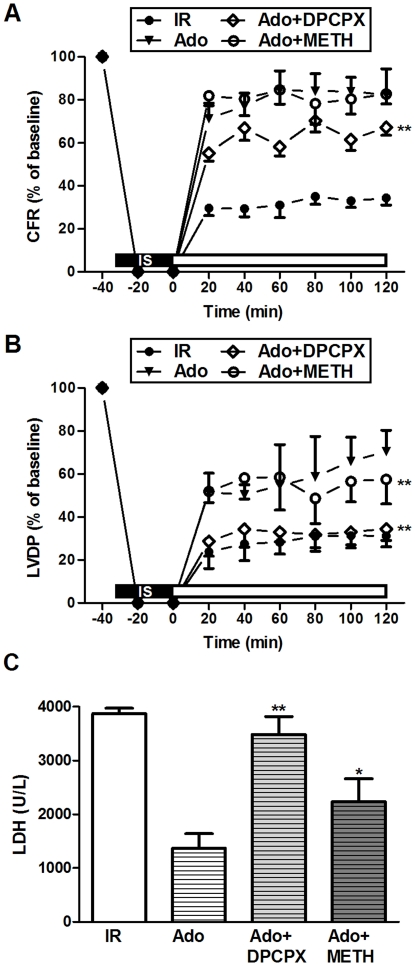
Effects of A_1_AR and M_2_AChR blockade on adenosine-mediated cardioprotection. (A) DPCPX abolished the increase in coronary flow rate (CFR) mediated by adenosine; METH had little effect on CRF. (B) DPCPX abolished the left-ventricular developed pressure (LVDP) improvement mediated by adenosine; METH dampened the effect on LVDP (C) DPCPX and METH suppressed the decrease in LDH release mediated by adenosine in coronary effluent. IR: ischemic reperfusion; Ado: adenosine; IS: iscehmia. ^*^ *P*<0.05, ^**^ *P*<0.01 *vs.* IR; ^#^ *P*<0.05,^ # #^ *P*<0.01 *vs.* Ado (n = 6 in each group).

Co-administration of the selective A_1_AR antagonist DPCPX (1.0 µM) and adenosine or the selective M_2_AChR antagonist METH (1.0 µM) and adenosine was performed. Our result showed that DPCPX blunted the coronary vasodilatation afforded by adenosine (*P*<0.01 *vs.* Ado group Fig. 4A); METH had little effect on CRF. Furthermore, DPCPX abolished adenosine-elicited protective effect on myocardial contractile recovery. Surprisingly, METH dampened the effect of adenosine on LVDP (*P*<0.01 *vs.* Ado group Fig. 4B). In addition, both DPCPX and METH suppressed the adenosine-mediated decrease in LDH release (3489±135 U/L for Ado+DPCPX *vs.* 1370±111 U/L for Ado group, *P*<0.01; 2235±175 U/L for Ado+METH *vs.* Ado group, *P*<0.05, Fig. 4C). Treatment with DPCPX or METH alone had no effect on cardiac function (data not shown). Thus, DPCPX nearly abolished the cardioprotection induced by adenosine during reperfusion. Intriguingly, METH was able to partially reverse the protective effects of adenosine.

### Effects of A_1_AR and M_2_AChR Blockade on Acetylcholine-mediated Cardioprotection

**Figure 5:**
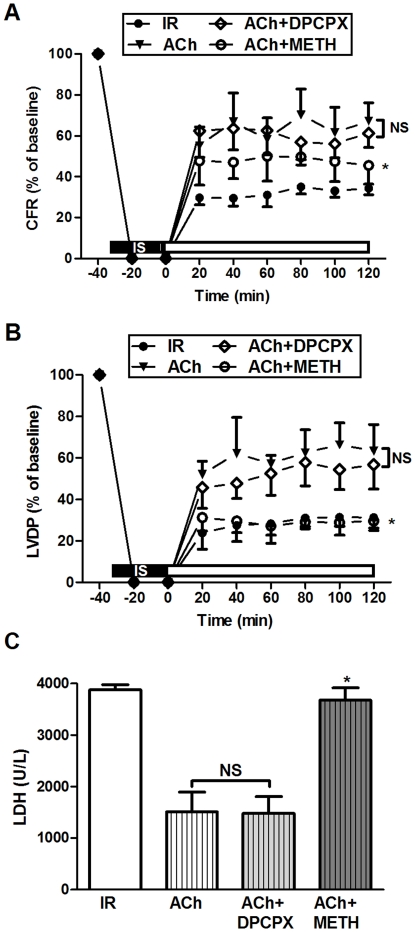
Effects of A_1_AR and M_2_AChR blockade on acetylcholine-mediated cardioprotection. (A) METH abolished the increase in coronary flow rate (CFR) mediated by acetylcholine; (B) METH abolished the left-ventricular developed pressure (LVDP) improvement mediated by acetylcholine; (C) METH abolished the decrease in LDH release mediated by acetylcholine in coronary effluent. IR: ischemic reperfusion; ACh: acetylcholine; IS: iscehmia. ^*^ *P*<0.05, ^**^ *P*<0.01 *vs.* IR; ^&^ *P*<0.05, ^&&^ *P*<0.01 *vs.* ACh; NS: not significance (n = 6 in each group).

Simultaneous treatment with the selective A_1_AR antagonist DPCPX (1.0 µM) and acetylcholine or the selective M_2_AChR antagonist METH (1.0 µM) and acetylcholine was performed. Our data revealed that DPCPX was unable to attenuate the acetylcholine-induced cardioprotection (*P*>0.05 *vs.* ACh group, Fig. 5). METH abolished the protective effects of acetylcholine, which abrogated the acetylcholine-mediated increase in CFR and LVDP (*P*<0.01 *vs.* ACh group, Fig. 5A, B), as well as the decrease in LDH release (*P*<0.01 *vs.* ACh group, Fig. 5C). Thus, acetylcholine participated in prevention of reperfusion injury mostly through M_2_AChR activation. DPCPX failed to inhibit its cardioprotection.

### Adenosine Increased the Protein Expression of M_2_AChR

**Figure 6:**
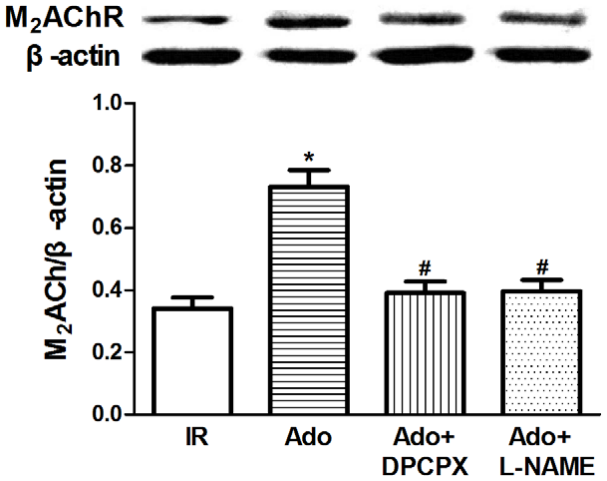
Effects of adenosine on the protein expression of M_2_AChR. Adenosine increased the protein level of M_2_AChR and DPCPX or L-NAME abolished this effect. IR: ischemic reperfusion; Ado: adenosine; ^*^ *P*<0.01 *vs.* IR; ^#^ *P*<0.01 *vs.* Ado (n = 6 in each group).

The protein expression of M_2_AChR was determined by Western blot. Adenosine treatment at reperfusion significantly increased the protein level of M_2_AChR compared with the IR group (*P*<0.01 *vs.* IR group, Fig. 6). Co-treatment with DPCPX or L-NAME abolished adenosine-induced increase in M_2_AChR protein expression (*P*<0.01 *vs.* Ado group). Treatment with either DPCPX or L-NAME alone exerted little effect on protein expression of M_2_AChR (data not shown). Thus, adenosine increased the protein level of M_2_AChR mainly through A_1_AR activation and NOS pathway may be involved in such effect.

### Adenosine Increased the *B*_max_ of MAChR

**Figure 7:**
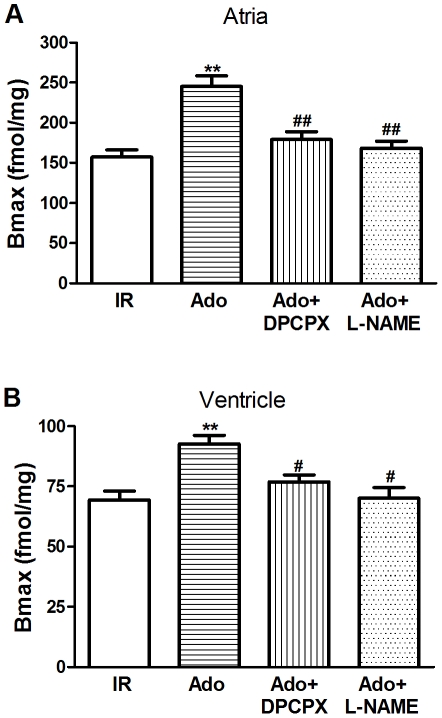
Effects of adenosine on the *B*_max_ of MAChR. Adenosine increased the *B*_max_ of MAChR both in atria (A) and in ventricle (B) of ischemic-reperfused myocardium. DPCPX or L-NAME abolished this effect. IR: ischemic reperfusion; Ado: adenosine; *P*<0.05, ^**^ *P*<0.01 *vs.* IR; ^#^ *P*<0.05, ^##^ *P*<0.01 *vs.* Ado (n = 6 in each group).

A radioligand-receptor binding assay was performed to evaluate the capacity of the muscarinic receptor. Scatchard analysis of the saturation curves demonstrated the density of muscarinic receptors by the maximal binding capacity (*B*_max_). Adenosine at reperfusion significantly increased the *B*_max_ (Ado group: 245±33 fmol/mg in atria and 93±9 fmol/mg in ventricle, *vs.* IR group: 157±22 fmol/mg in atria and 69±9 fmol/mg in ventricle, *P*<0.01, Fig. 7). Both DPCPX and L-NAME inhibited adenosine-induced *B*_max_ improvement (*P*<0.01 *vs.* Ado group in atria; *P*<0.05 *vs.* Ado group in ventricle). These results were consistent with the data from the M_2_AChR protein expression. Treatment with DPCPX or L-NAME alone had no effects on *B*_max_ of MAChR (data not shown). Therefore, adenosine increased the *B*_max_ of MAChR mainly through A_1_AR activation and NOS pathway may be involved in such effects.

## Discussion

The present experiments showed that both adenosine and acetylcholine ameliorated cardiac function in the reperfused rat heart. Simultaneous administration of adenosine and acetylcholine failed to offer any additional beneficial effect. Further, we found that NOS might act in a shared pathway with a common converging point. Intriguingly, selective M_2_AChR antagonist partially attenuated the cardioprotective effect of adenosine. Otherwise, adenosine contributed to the invigorating effect on muscarinic receptors. These data depicted a possible functional coupling between the adenosine and muscarinic receptors in mediating cardioprotection in the rat heart.

It has been convincingly reported that A_1_AR is essential to the adenosine-induced cardioprotection against reperfusion injury. Administration of A_1_AR blocker DPCPX abolishes the infarct-limiting effect in isolated rabbit hearts following global ischemia-reperfusion [5]. The finding is further supported in animal models with a complete loss of cardioprotection in A_1_AR knock-out mice [15]. In addition, activation of A_1_AR by endogenous adenosine during reperfusion has also been shown to be protective in mouse hearts [16]. In agreement with previous studies, an obvious improvement in cardiac function is achieved by adenosine in reperfused rat hearts, and the A_1_AR antagonist DPCPX abolishes this effects. On the other hand, it is more novel that the M_2_AChR antagonist METH suppresses the adenosine-induced cardioprotection partly.

Several lines of evidence suggest that adenosine is cardioprotective in patients with congestive heart failure and ischemic heart disease via attenuation of catecholamine release and metabolic responses to β-adrenergic stimulation, which is mainly mediated by A_1_AR [17]. Pelleg and colleagues elucidated the anti-adrenergic actions of adenosine both in vivo and in vitro. In the presence of isoproterenol (0.2 mg/kg/min), adenosine (0.01–0.1 µM/kg) given as a rapid bolus potentiated the significantly negative chronotropic action in rats. Similarly, in isolated perfused hearts, adenosine administration attenuated isoproterenol-induced chronotropic and inotropic effects [18]. In contrast, our previous study showed that adenine sulfate (the exogenous precursor of adenosine) reduced cardiac dysfunction following coronary ligation accompanied by increasing the distribution density of cholinesterase-positive nerves and the expression of M_2_AChR in post-infarction rat hearts [11]. In our present study, we identified the adenosine and muscarinic receptor interaction in rat hearts. Our data further revealed that activation of either adenosine or muscarinic receptor resulted in a significant improvement in cardiac function against reperfusion injury (Fig. 1), which was abolished by antagonism against either A_1_AR or M_2_AChR (Fig. 4). Of particular interest is the observation that the M_2_AChR antagonist METH also blunts the adenosine-induced cardioprotection (i.e., increase in LVDP and decrease in LDH), although it failed to antagonize the coronary vasodilatory effect (Fig. 4). To the contrary, the A_1_AR antagonist DPCPX had no effects on acetylcholine-mediated protection (Fig. 5). Furthermore, adenosine treatment overtly elevated protein expression (Fig. 6) and *B*_max_ of MAChR (Fig. 7), which was abolished by the selective A_1_AR antagonist DPCPX. These data represent the first piece of evidence on a cross-talk between adenosine and muscarinic receptors.

Simultaneous administration of both adenosine and acetylcholine failed to exert an additive protective effect over either agonist alone, suggesting a shared mechanism with a possible converging check-point (Fig. 1). Indeed, it appears that A_1_AR and M_2_AChR activation may act in concert to afford cardioprotection. It has been demonstrated that adenosine can induce NO release in a wide variety of cells including endothelial cells, smooth muscle cells and cardiomyocytes. Adenosine increases NO production through activation of adenosine receptors and NOS. NO has been known to play a crucial role in the protective effect of adenosine on mitochondrial oxidant damage [19]. Likewise, Yao and Gross examined the involvement of NOS in the anti-infarct effect of acetylcholine in the canine heart, while their results indicated that acetylcholine-elicited protection may be blocked by L-NAME [20]. In this study, we found that adenosine and acetylcholine independently increased NO release and rescued myocardial reperfusion injury. This effect was blocked by the appropriate receptor antagonists as well as the NOS inhibitor L-NAME, indicating a pivotal role of NO as a distal effector for cardioprotection (Fig. 2, 3). Taken together, the data presented in this study implicates an integral role for the NO in the signal pathway following adenosine and muscarinic receptor activation.

In our present experiment, adenosine treatment improved muscarinic receptors and contractile function in reperfused myocardium. Based on data from our current study and others, it is plausible to speculate that adenosine-mediated cardioprotection is associated with not only an anti-adrenergic effect, but also elevated parasympathetic tone. This amelioration of cardiovascular function may be a potentially novel mechanism of adenosine.
